# New Lignans and Iridoid Glycosides from *Dipsacus asper* Wall

**DOI:** 10.3390/molecules20022165

**Published:** 2015-01-28

**Authors:** Xinguang Sun, Guoxu Ma, Dawei Zhang, Wenhua Huang, Gang Ding, Huagang Hu, Guangzhong Tu, Baolin Guo

**Affiliations:** 1 Key Laboratory of Bioactive Substances and Resources Utilization of Chinese Herbal Medicine, Ministry of Education, Institute of Medicinal Plant Development, Chinese Academy of Medical Sciences and Peking Union Medical College, Beijing 100193, China; E-Mails: sxgzhwu07@163.com (X.S.); mgxfl8785@163.com (G.M.); zhangdw517@gmail.com (D.Z.); whhuang@implad.ac.cn (W.H.); 2 State Nationalities Affairs Commission and Department of Educational Key Lab of Minority Traditional Medicine, MINZU University of China, Beijing 10081, China; E-Mail: tangang_666@163.com; 3 Beijing Institute of Microchemistry, Beijing 100091, China; E-Mail: tgz2008@microchem.org.cn

**Keywords:** *Dipsacus asper* Wall, lignans, bis-iridoid glycoside

## Abstract

Six new compounds, including four new lignans, dipsalignan A (**1**), B–D (**3**–**5**), and two new bis-iridoid glycoside dimmers, dipsanosides M (**7**) and N (**8**), together with two known compounds (**2**) and (**6**), have been isolated from the roots of *Dipsacus asper* Wall. Their structures were established on the basis of spectroscopic data (MS, 1D, 2D NMR, and CD) and chemical methods. All the isolated compounds were tested against human immunodeficiency virus-1 (HIV-1) integrase inhibition activities, and only compounds **1**, **2**, **7**, and **8** displayed weak activities.

## 1. Introduction

**Figure 1:**
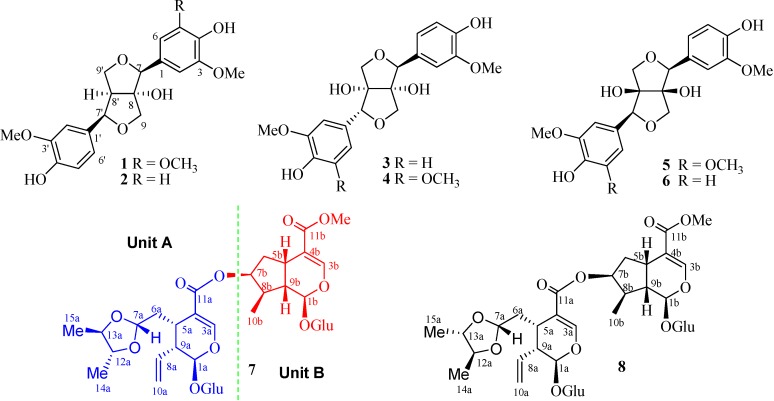
Chemical Structures of Compounds **1**–**8**.

*Dipsacus asper* Wall. is a perennial plant widely distributed in south China. Its dried root, named “Xuduan” in Chinese, has been used as a tonic for refreshment, a fissiparism promoter of the osseous cells, and as an anti-aging agent in Traditional Chinese Medicine (TCM) for hundreds of years. Additionally, *D. asper* can also act as antibacterial, anti-inflammatory, and immunomodulatory agents [1,2,3], and it also can be used for the treatment of postmenopausal osteoporosis [4]. Our previous investigation on bioactive constituents from the roots of *Dipsacus asper* led to the isolation of new triterpene glycosides [5]. In our ongoing investigation of secondary metabolites from this medicinal plant, six new compounds, including four new lignans, dipsalignan A (**1**), B–D (**3–5**), and two new bis-iridoid glycoside dimmers, dipsanosides M (**7**) and N (**8**), together with two known compounds, (+)-1-hydroxy-2,6-bis-*epi*-pinoresinol (**2**) [6] and (+)-1, 5-dihydroxy-2(*S*), 6(*S*)-di(4-hydroxy-3-methoxyphenyl)-3,7-dioxabicyclo[3.3.0] octane (**6**) [7] (Figure 1), were isolated. Their structures were elucidated by extensive analysis of HR-ESI-MS, 1D and 2D NMR, CD data, and chemical methods. In this paper, we report the isolation, structural elucidation of the new compounds, and their inhibitory activities against human immunodeficiency virus (HIV-1) integrase.

## 2. Results and Discussion

**Figure 2:**
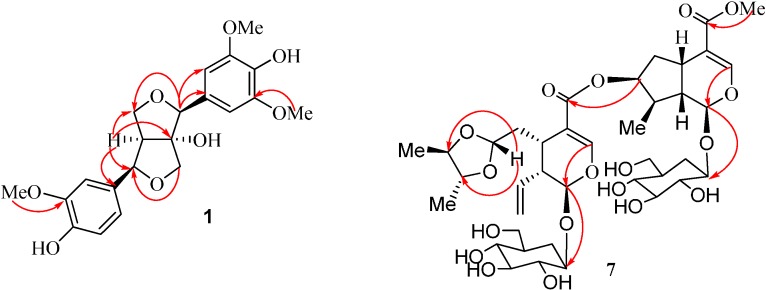
The key HMBC correlations of Compounds **1** and **7**.

Compound **1** was obtained as a white powder and its molecular formula was determined to be C_21_H_24_O_8_ by high resolution (HR)-ESI-MS analysis (*m*/*z* 427.1388 ([M+Na]^+^, calcd: 427.1383) Absorptions in the IR spectrum were attributed to the hydroxyl functions (3412 cm^−1^) as well as aromatic ring (1602, 1516 cm^−1^). The ^1^H-NMR spectra (Table 1) showed the presence of a 1,3,4,5-tetrasubstituted phenyl group [δ_H_ 6.75 (2H, d, *J* = 1.8 Hz)] and a 1,3,4-trisubstituted phenyl group [δ_H_ 7.07 (1H, d, *J* = 1.8 Hz), 6.85 (1H, dd, *J* = 7.8, 1.8 Hz), 6.77 (1H, d, *J* = 7.8 Hz)], and three methoxyl groups [δ_H_ = 3.85 (6H, s), 3.87 (3H, s)]. Additionally, it also showed two methine units (two of which oxygenated) at δ_H_ 4.65 (s), 4.43 (d, *J* = 7.8 Hz), 2.64 (1H, dd, *J* = 7.8, 6.6 Hz), two oxygenated methylene signals at δ_H_ 3.42 (1H, d, *J* = 9.0 Hz), 3.65 (1H, d, *J* = 9.0 Hz); δ_H_ 4.01 (1H, d, *J* = 9.0 Hz), 4.07 (1H, dd, *J* = 9.0, 6.6 Hz). The ^13^C-APT spectra of **1** (Table 1) exhibited 21 carbons including 12 aromatic carbons, three methoxyls, two oxygenated methines, two oxygenated methylenes, one methine and one oxygenated quaternary carbon, which were consistent with the ^1^H NMR spectra. Comparing the NMR spectra of the known (+)-8-hydroxy-7,7'-bis-*epi*-pinoresinol (**2**) [6] with those of **1** revealed the similar structural fragments present in those two compounds except for one more additional methoxyl group observed in **1**. The HMBC experiment confirmed the above-mentioned suggestion and put the additional methoxyl unit at C-5 (Figure 2). Thus the planar structure of **1** was established.

**Table 1:** ^1^H- and ^13^C-NMR data for **1**, and **3**–**5** (600 and 125 MHz, δ ppm).

NO.	1 (CD3OD)		3 (CDCl_3_)		4 (CDCl_3_)		5 (CDCl_3_)	
	δ_H_ (*J*)	δ_C_	δ_H_ (*J*)	δ_C_	δ_H_ (*J*)	δ_C_	δ_H_ (*J*)	δ_C_
1	—	129.6	—	128.6	—	129.3	—	129.3
2	6.75 (s)	103.9	6.92 (d, 1.8)	109.2	6.97 (d, 1.8)	112.3	6.93 (d, 1.8)	109.9
3	—	149.3	—	147.2	—	146.9	—	147.1
4	—	136.2	—	145.7	—	145.7	—	145.7
5	—	149.3	6.76 (dd, 7.8, 1.8)	115.2	6.69 (dd, 7.8, 1.8)	114.7	6.77 (dd, 7.8, 1.8)	115.1
6	6.75 (s)	103.9	6.81 (d, 7.8)	117.5	6.77 (d, 7.8)	120.2	6.80 (d, 7.8)	118.1
7	4,65 (s)	87.4	4.53 (s)	85.3	4.53 (s)	85.4	4.66 (s)	84.6
8	—	94.4	—	88.2	—	88.2	—	84.7
9α	3.42 (d, 9.0)	77.4	3.51 (d,9.0)	74.3	3.54 (d, 9.0)	74.5	3.39 (d,9.0)	72.9
9β	3.65 (d, 9.0)		3.19 (d,9.0)		3.20 (d, 9.0)		3.68 (d,9.0)	
1'	—	133.5	—	127.7	—	128.4	—	128.4
2'	7.07 (d, 1.8)	111.3	6.97 (d, 1.8)	112.2	6.93 (d, 1.8)	102.5	6.64 (d, 1.8)	103.2
3'	—	149.5	—	146.8	—	147.8	—	147.7
4'	—	148.0	—	145.9	—	134.7	—	134.7
5'	6.77 (d, 7.8)	116.2	—	115.2	—	147.8	—	147.7
6'	6.85 (dd, 7.8, 1.8)	120.8	6.78 (d, 1.8)	120.2	6.93 (d, 1.8)	102.5	6.64 d, 1.8)	103.2
7'	4.43 (d, 7.8)	90.7	4.30 (s)	88.9	4.30 (s)	88.9	4.66 (s)	84.5
8'	2.64 (dd, 7.8, 6.6)	64.1	—	85.3	—	85.4	—	84.7
9'α	4.01 (dd, 9.0, 1.2)	70.7	4.18 (d,9.0)	75.5	4.18 (d, 9.0)	74.5	3.68 (d, 9.0)	73.0
9'β	4.07 (dd, 9.0, 1.2)		3.43 (d,9.0)		3.44 (d, 9.0)		3.39 (d, 9.0)	
3-OMe	3.85 (s)	56.9	3.76 (s)	55.6	3.76 (s)	55.6	3.76 (s)	55.5
5-OMe	3.85 (s)	56.9	—	—	—	—	—	—
3'-OMe	3.87 (s)	56.6	3.76 (s)	55.6	3.76 (s)	56.0	3.76 (s)	55.9
5'-OMe	—	—	—	—	3.76 (s)	56.0	3.76 (s)	55.9

The relative configuration of **1** was determined by analysis of the coupling constants and comparison of the compound **2**. The H-7' possessed a α-orientation on the basis of the observed *J* value of 7.8 Hz between H-7' and H-8', indicating that H-7' and H-8' were in a *cis*-configuration [8]. H-7 was also placed in the α-orientation based on the upfield shifts of H-9α (δ_H_: 3.42 ppm) and H-9β (δ_H_: 3.65 ppm) due to shielding by the phenyl ring at C-7 in **1** compared with the chemical shift of H-9α (δ_H_: 3.84 ppm) and H-9β (δ_H_: 3.90 ppm) in **2** [9]. The NOESY correlations from H-8' to H-7' and H-7 further supported the conclusion. Thus the relative configuration for **1** was determined. The CD data (positive contton effects at 235, and negative ones at 216 nm) and optical rotation {${\left[\text{α}\right]}_{D}^{20}$ = 28.2° (*c* = 0.060, MeOH)} of **1** were similar to those of **2** [6], which indicated that **1** and **2** had same absolute configurations. Consequently, the structure of **1** was defined as (+)-8-hydroxy-7,7'-bis-*epi*- fraxiresinol, and was given the trivial name dipsalignan A.

Compound **3** was obtained as a white powder. Its molecular formula was established as C_20_H_22_O_8_ derived from HR-ESI-MS analysis (*m*/*z* 413.1217 ([M+Na]^+^, calcd: 413.1212). The IR spectra showed the hydroxyl functions (3401 cm^−1^) and aromatic ring (1616, 1516 cm^−1^). The ^1^H-NMR spectra of **3** (Table 1) showed the presence of two ABX system aromatic rings [δ_H_ 6.92 (d, *J* = 1.8 Hz), 6.76 (1H, dd, *J* = 7.8, 1.8 Hz), 6.81 (1H, d, *J* = 7.8 Hz); δ_H_ 6.97 (1H, d, *J* = 1.8 Hz), 6.69 (1H, dd, *J* = 7.8, 1.8 Hz), 6.78 (1H, d, *J* = 7.8 Hz)], two methoxyl groups (δ_H_ = 3.78, 3.83). The ^13^C-APT spectrum of **3** (Table 1) exhibited 20 carbons including 14 olefinic carbons, two methoxyl groups, two oxygenated methines, and two oxygenated methylenes. Detailed analysis of the NMR data especially the 2D NMR experiments confirmed that compound **3** possessed the same planar structure as that of the known compound prinsepiol [7], indicating of the 8,8' -dihydroxy-7,9':7',9-diepoxylignan structure in **3** [10]. The relative configuration of **3** was established by following analysis. The upfield shift of H-7' (δ_H_: 4.30 ppm) due to the shielding by the phenyl ring at C-7 compared to the chemical shift of H-7 (δ_H_: 4.53) in **3**, suggesting that benzyl group of **3** at C-7 was placed in the β-orientation [11,12,13]. Moreover, the downfield shift of C-7' (δ_C_: 88.9 ppm) and upfield shifts of H-9α (δ_H_: 3.19 ppm) and H-9β (δ_H_: 3.51 ppm) due to shielding by the phenyl ring at C-7 in **3**, which also supported that the phenyl ring at C-7 in **3** possessed the β-orientation, and H-7 and H-7' were *threo*-configuration [9,11,12,13]. In addition, the specific rotation of **3** was consistent with the report of vitexkarinol [14], but opposite to that of *epi*-pinoresionl-4, 4'-di-*O*-β-d-glucopyranoside [15]. On the basis of the above evidences, compound **3** was determined as (+)-(7*S*, 8*S*, 7'*R*, 8'*S*)-prinsepiol, and named dipsalignan B.

Compound **4** gave molecular formula C_21_H_24_O_9_ from HR-ESI-MS analysis (*m*/*z* 443.1317 ([M+Na]^+^, calcd: 443.1313). The NMR spectra of **4** (Table 1) was similar to those of **3** except for the 1,3,4-trisubstituted phenyl group in **3** was replaced by 1,3,4,5-tetrasubstituted phenyl group in **4**, and one additional methoxy unit was present in compound **4**. In the HMBC spectra, the additional methoxy group in **4** had the correlation with C-5' putting the methoxyl anchor at C-5', which established the structure of **4**. Thus, compound **4** was determined as (+)-(7*S*, 8*S*, 7'*R*, 8'*S*)-5-methoxyprinsepiol, and given the trivial name of dipsalignan C.

The molecular formula of compound **5** was assigned as C_21_H_24_O_9_ on the basis of the HR-ESI-MS *m*/*z* 443.1336 ([M+Na]^+^, calcd: 443.1313). Its NMR spectroscopic data especially 2D NMR correlations of **5** characterized the planar structure actually same as that the known compound, (7*R*, 8*S*, 7'*R*, 8'*S*)-5-methoxyprinsepiol [16], which possessed the 8,8'-dihydroxy-7, 9':7', 9-diepoxylignan structural features. The completely opposite CD data and optical rotation degrees compared with the known compounds revealed the absolute configuration of **5** as (+)-(7*S*, 8*R*, 7'*S*, 8'*R*)-5-methoxyprinsepiol [16], and compound **5** was given the trivial name of dipsalignan D.

**Figure 3:**
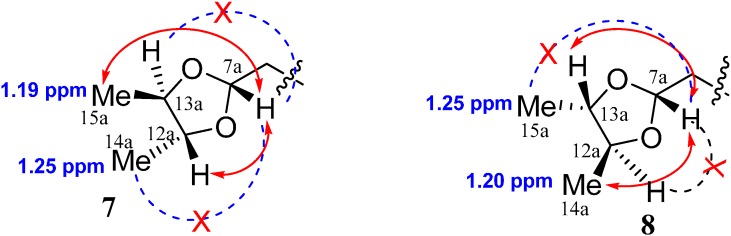
The NOESY correlations in **7** and **8**.

Compound **7** was obtained as a white powder and its molecular formula was determined to be C_37_H_54_O_20_ by HR-ESI-MS analysis (*m*/*z* 841.3096 ([M+Na]^+^, calcd: 841.3130) Two distinct moieties indicated that unit A and unit B could be identified by comparing the ^1^H and ^13^C NMR spectra of **7** (Table 2) to the known compound [17,18], and these two units were connected via an ester bond between C-11a and C-7b, which was confirmed by HMBC correlation from H-7b to C-11a. The characteristic anomeric protons of sugar at δ 4.69 (1H, d, *J* = 7.8 Hz) and δ 4.69 (1H, d, *J* = 7.8 Hz) and anomeric carbon signals at δ_C_ 100.4 and 97.7 in the ^1^ H and ^13^ C NMR spectra of **7** collectively suggested the existence of two sugar units in **7**. Furthermore, acid hydrolysis of **7** yielded d-glucose, which was identified by GC analysis. The HMBC correlation from H-1'a to C-1a, and from H-1'b to C-1b, supported that two D-glucoses were at position C-1a of unit A and C-1b of unit B, respectively (Figure 2). The NMR spectra of **7** were almost identical to those of dipsanoside C [19] except for an additional presence of one 4,5-dimethyl-1,3-dioxolane moiety in **7**, which was further confirmed by HMBC correlations from H-7a to C-12a and C-14a, H-12a to C-14a, and H-13a to C-14a and C-15a. The relative configurations of the 4, 5-dimethyl-1, 3-dioxolane moiety in **7** were determined based on NOESY correlations. In the NOESY spectra, the clear cross peak from H-7a to CH_3_-15a confirmed that H-7a and CH_3_-15a were on the same side of 1,3-dioxolane ring system, whereas the absent correlation between H-7a and CH_3_-14a suggested that this methyl group was on the opposite face of the corresponding ring (Figure 3). Consequently, the structure of **7** was determined as dipsanoside M.

Compound **8** was also obtained as white amorphous powder. Its molecular formula, C_37_H_54_O_20_, was the same as those of compound **8**, indicating **8** to be an isomer of **7**. The NMR spectra especially 2D NMR including HMBC and NOESY of **8** (Table 2) confirmed that compound **8** possessed the same planar structure as that of **7** except for the configuration of the 4, 5-dimethyl-1,3-dioxolane moiety. The NOESY correlations from H-7a to CH_3_-14a suggested that H-7a and CH_3_-14a were orientated on the same side of the 1,3-dioxolane ring, whereas the absent correlations between CH_3_-15a and H-7a implied that the 15a-methy was on the other side of the ring (Figure 3). Thus the structure of 8 was determined named as dipsanosides N.

Compounds **1**–**8** were assayed for their HIV-1 integrase inhibition activities with a microplate screening method [20], compounds **1**, **2**, **7**, and **8** showed inhibition activities against HIV-1 integrase with IC_50_ values in 53.26 µM, 61.74 µM, 84.03 µM, and 92.67 µM, respectively, together with IC_50_ value of positive control, baicalein in 1.37 µM.

**Table 2:** ^1^H-NMR (600 MHz) and ^13^C-NMR (125 MHz) data of **7** and **8** in CD_3_OD.

NO.	7		8	
	δ_H_, mult. (*J* in Hz)	δ_C_	δ_H_, mult. (*J* in Hz)	δ_C_
1a	5.54 (d, 6.0)	98.0	5.55 (d, 6.0)	98.0
3a	7.44 (s)	153.5	7.44 (s)	153.6
4a	—	113.6	—	113.6
5a	3.02 (br q, 6.0)	30.2	3.01 (br q, 6.0)	30.1
6a	1.73–1.80 (m) 2.02 (ddd, 6.0, 6.0, 14.0)	35.9	1.73–1.82 (m) 1.99 (ddd, 6.0, 6.0, 14.0)	36.0
7a	5.13 (dd, 4.2, 6.0)	103.4	5.13 (dd, 4.2, 6.0)	103.3
8a	5.75 (ddd, 8.4, 10.2, 18.0)	136.0	5.75 (ddd, 8.4, 10.2, 18.0)	136.0
9a	2.75 (ddd,5.4, 6.0, 8.4)	45.5	2.75 (ddd, 5.4, 6.0, 8.4)	45.6
10a	5.29 (d, 18.0) 5.26 (d, 10.2)	120.0	5.29 (d, 18.0) 5.25 (d, 10.2)	120.0
11a	—	168.6	—	168.6
12a	3.56 (d,6.0)	61.4	3.56 (d, 3.5)	81.2
13a	3.56 (d,6.0)	79.4	3.56 (d, 3.5)	79.6
14a	1.19 (d,6.0)	17.5	1.20 (d, 6.0)	17.6
15a	1.25 (d,6.0)	17.6	1.25 (d, 6.0)	17.7
1'a	4.69 (d,7.8)	100.4	4.69 (d, 7.8)	100.4
2'a	3.20 (dd,7.8,9.0)	75.0	3.20 (dd, 7.8, 9.0)	75.0
3'a	3.25–3.39 (m)	78.2	3.25–3.39 (m)	78.2
4'a	3.25–3.39 (m)	71.9	3.25–3.39 (m)	71.9
5'a	3.25–3.39 (m)	78.6	3.25–3.39 (m)	78.6
6'a	3.66 (dd, 6.0, 12.0) 3.88–3.92 (m)	63.0	3.67 (dd, 6.0, 12.0) 3.91 (br d, 12.0)	63.0
1b	5.27 (d, 4.2)	97.7	5.27 (d, 4.2)	97.6
3b	7.42 (s)	152.6	7.42 (s)	152.6
4b	—	112.2	—	112.2
5b	3.13 (br q, 7.8)	32.8	3.12 (br q, 7.8)	32.7
6b	1.73–1.80 (m) 2.32 (br dd, 7.8, 14.4)	40.6	1.74–1.82 (m) 2.32 (br dd, 7.8, 14.4)	40.5
7b	5.20 (dd, 4.2, 7.8)	78.6	5.19 (dd, 4.2, 7.8)	78.6
8b	2.09–2.16 (m)	41.2	2.10–2.16 (m)	41.1
9b	2.09–2.16 (m)	47.4	2.10–2.16 (m)	47.4
10b	1.07 (d, 6.5)	14.0	1.07 (d, 6.0)	13.9
11b	—	169.5	—	169.5
12b	3.69 (s)	51.9	3.69 (s)	51.9
1'b	4.67 (d, 7.8)	100.3	4.67 (d, 7.8)	100.3
2'b	3.20 (dd, 7.8, 9.0)	74.9	3.20 (dd, 7.8, 9.0)	74.9
3'b	3.25–3.39 (m)	78.2	3.25–3.39 (m)	78.2
4'b	3.25–3.39 (m)	71.8	3.25–3.39 (m)	71.8
5'b	3.25–3.39 (m)	78.6	3.25–3.39 (m)	78.6
6'b	3.66 (dd, 6.0, 12.0) 3.88–3.92 (m)	63.0	3.66 (dd,6.0,12.0) 3.89 (br d,12.0)	63.0

## 3. Experimental Section

### 3.1. General

Optical rotations were obtained on a Perkin-Elmer 341 digital polarimeter (Perkin-Elmer Corp., Waltham, MA, USA). IR and UV spectra were recorded on FTIR-8400S spectrometer (Shimadzu Corp., Tokyo, Japan) and Shimadzu UV2550 (Shimadzu Corp, Tokyo, Japan), respectively. CD spectra were recorded on a JASCO J-815 spectropolarimeter (JASCO Corp., Tokyo, Japan). NMR spectra were obtained with a Bruker AV III 600 NMR spectrometer (Bruker Corp., Munich, Germany) (chemical shift values are presented as δ values with TMS as the internal standard; Munich, German). HR-ESI-MS spectra were performed on a LTQ-Obitrap XL spectrometer (Thermo Fisher Scientific Inc., Waltham, MA, USA). GC analysis was carried out on a GC-7890: column, DB-5 (30 m × 0.32 mm, 0.25 mm); detector, FID-6850 (Agilent, Santa Clara, CA, USA). ODS gel (50 µm, YMC, Kyoto, Japan), Sephadex LH-20 (Pharmacia, Uppsala, Sweden), and MCI gel (CHP 20P, 75–150 mm, Mitsubishi Chemical Corporation, Tokyo, Japan) were used for column chromatography. MPLC separations were carried out on an Agela CHEETAH series. HPLC separations were performed using a Waters 2535 series pump equipped with a PDA detector and a YMC (250 × 10 mm, 5 μm) preparative column. TLC was carried out on silica gel GF254 (Yantai Chemical Inst., Yantai, China) plates, and spots were visualized under UV light (254 or 356 nm) or by spraying with 5% H_2_SO_4_ in 95% EtOH followed by heating.

### 3.2. Plant Material

The roots of *D. asper* were collected from Guizhou province in July 2011, and was authenticated by one of the authors (B.-L. Guo). A voucher specimen is deposited at Natural Medicine Research Center of Institute of Medicinal Plant Development, Chinese Academy of Medical Science and Peking Union Medical College.

### 3.3. Extration and Isolation

Dried roots (10 kg) of *D. asper* were cut into small pieces and refluxed with 75% (*v*/*v*) EtOH (120 L × 2 h, 3 times). The residue was separated by column chromatography on macroporous resin D101 and eluted with water, 30%, 50% and 95% ethanol in successive. The 30% ethanol eluate was separated over a MCI gel column eluted with a MeOH–H_2_O (10:90 to 100:0, *v*/*v*) gradient system to give five fractions (Fr. A–E). Fr. B (55 g) was subjected to column chromatography over reversed-phase C_18_ silica gel eluted with MeOH–H_2_O (15:85 to 40:60, *v*/*v*) to give five subfractions (Fr. B_1_ to Fr. B_5_). Fr. B_1_ (3 g) was subjected to RP flash CC (15%–20% MeOH in H2O) to give three subfractions Fr. B_1–1_ to Fr. B_1–3_. Fr. B_1–3_ with Sephadex LH-20 followed RP semipreparative HPLC (ACN–H_2_O, 18:82, *v*/*v*) to afford **1** (11 mg) and **2** (18 mg). Fr. B_2_ (5 g) was fractioned by RP flash chromatography (15%–20% MeOH in H_2_O) to give four subfractions Fr. B_2–1_ to Fr. B_2–4_. The subfraction Fr. B_2–3_ was further purified successively by chromatography over Sephadex LH-20 and RP semipreparative HPLC (ACN–H_2_O, 23:77, *v*/*v*) to yield **3** (13 mg), **4** (4 mg), **5** (6 mg), and **6** (5 mg). Fr. C (10 g) was separated by a column of RP silica gel eluted with MeOH–H_2_O (20:80 to 80:20, *v*/*v*) to give three subfractions (Fr. C_1_ to Fr. C_3_). The subfraction Fr. C_3_ (5 g) was separated by RP flash CC (MeOH–H_2_O, 25:75 to 50:50, *v*/*v*) and purified by preparative HPLC using CH_3_CN–H_2_O (25:75, *v*/*v*) as mobile phase to afford **7** (6 mg) and **8** (7 mg).

### 3.4. Spectroscopic Data

Compound **1**, white amorphous powder; ${\left[\text{α}\right]}_{D}^{20}$ = 28.2° (*c* = 0.060, MeOH); UV λ_max_ (MeOH) nm: 211, 236, and 280 nm; CD nm (Δε) (c 0.001, MeOH): 235 (0.59), 216 (−1.27); IR (KBr) cm^−1^: 3391, 1603, 1514. ^1^H- and ^13^C-NMR data, see Table 1; HR-ESI-MS *m*/*z* 427.1388 [M+Na]^+^, (calcd: 427.1382).

Compound **3**, white amorphous powder; ${\left[\text{α}\right]}_{D}^{20}$ = 21.6° (*c* = 0.065, MeOH); UV λ_max_ (MeOH) nm: 204, 231, and 280 nm; IR (KBr) cm^−1^: 3401, 1616, 1516. ^1^H- and ^13^C-NMR data, see Table 1; HR-ESI-MS *m*/*z* 413.1217 [M+Na]^+^, (calcd for 413.1212).

Compound **4**, white amorphous powder; ${\left[\text{α}\right]}_{D}^{20}$ = 35.8° (*c* = 0.063, MeOH); UV λ_max_ (MeOH) nm: 204, 231, and 280 nm; IR (KBr) cm^−1^: 3397, 1614, 1513. ^1^H- and ^13^C-NMR data, see Table 1; HR-ESI-MS *m*/*z* 443.1317 [M+Na]^+^, (calc for 443.1313).

Compound **5**, white amorphous powder; ${\left[\text{α}\right]}_{D}^{20}$ = 71.2° (*c* = 0.053, MeOH); UV λ_max_ (MeOH) nm: 208, 230, and 279 nm; CD nm (Δε) (*c* 0.001, MeOH):, 278 (+0.96), 240 (+5.6); IR (KBr) cm^−1^: 3398, 1611, 1517. ^1^H- and ^13^C-NMR data, see Table 1; HR-ESI-MS *m*/*z* 443.1338 [M+Na]^+^, (calcd for 443.1313).

Compound **7**, white amorphous powder; ${\left[\text{α}\right]}_{D}^{20}$ = −31.6° (*c* = 0.053, MeOH); UV λ_max_ (MeOH) nm: 237 nm; IR (KBr) cm^−1^: 3425, 1697, 1637. ^1^H- and ^13^C-NMR data, see Table 2; HR-ESI-MS *m*/*z* 841.3096 [M+Na]^+^, (calcd for 841.3130).

Compound **8**, white amorphous powder; ${\left[\text{α}\right]}_{D}^{20}$ = −28.2° (*c* = 0.053, MeOH); UV λ_max_ (MeOH) nm: 237 nm; IR (KBr) cm^−1^: 3425, 1697, 1637. ^1^H- and ^13^C-NMR data, see Table 2; HR-ESI-MS *m*/*z* 841.3082 [M+Na]^+^, (calcd for 841.3130).

### 3.5. Acid Hydrolysis of Compounds ***8*** and ***9***

Compounds **8** (2.5 mg) and **9** (2.3 mg) were dissolved in 2N CF_3_COOH (5 mL) and heated at 95 °C for 6 h. After extraction three times with CH_2_Cl_2_ (5 mL), the remaining aqueous layer was repeatedly evaporated to dryness with EtOH until neutral. Then it was dissolved in anhydrous pyridine (2 mL), and added l-cysteine methyl ester hydrochloride (6 mg). After that, the mixture was stirred at 60 °C for 1 h. Furthermore, hexamethyldisilazane and trimethylchlorosilane (2:1; 3 mL) were added and kept at 60 °C for another 0.5 h [21]. Finally, the supernatant was analysed by GC under the following conditions: Agilent 6890N GC system; FID detector; HP-5 capillary column; column temperature: 180–250 °C, programmed increase, 15 °C/min; carrier gas: N_2_ (1 mL/min); injection temperature: 250 °C; injection volume: 1.0 μL, split ratio: 1/20. Consequently, the d-configurations of glucose, was confirmed by comparing with authentic sample, which showed retention times of 8.01 and 8.86 min, respectively.

### 3.6. HIV-1 Integrase Strand Transfer Inhibition Assays

The assays [20] were performed in a 96-well microplate (Corning, New York, NY, USA) in a final volume of 50 μL. The wells were washed once with the reaction buffer (25 mM/L PIPES, pH 7.0, 10 mM/L β-mercaptoethanol, 5% (*w*/*v*) glycerol, 0.1 g/L bovin serum albumin (BSA), and 10 mM/L MnCl_2_). The compounds were diluted with dimethyl sulfoxide (DMSO) to a final concentration of 10% DMSO into the reaction volume (*v*/*v*) and pre-incubated with 15 pmol integrase (IN) at 37 °C in the reaction buffer in the absence of MnCl_2_ (10 mM/L) for 10 min. Subsequently, 1.5 pM donor DNA and 15 pM target DNA were added and the reaction was initiated. After incubation for 1 h at 37 °C, 1.5 mL magnetic particles (6.7 × 10^8^ beads/mL) and 51.5 μL binding buffer (10 mM/L Tris–HCl, pH 7.6, 2 M/LNaCl, 20 mM/L ethylene diamine tetraacetic acid (EDTA), and 0.1% [*w*/*v*] Tween 20) were added and incubated at 20 °C for 15 min. Then the wells holding the mixture were placed in a magnetic concentrator, the supernatant was discarded, and the wells were washed 3 times with phosphate buffered saline (PBS) containing 0.1% Tween20(PBST). Subsequently, 100 μL of 1:5000 diluted alkaline phosphatase (AP) conjugate anti-DIG antibody was added and incubated for 30 min at 37 °C. Finally, the wells were washed 3 times with PBST and the magnetic beads were transferred into fresh wells; 100 μL P-nitrophenyl phosphate (P-NPP) substrate (0.1 M/L Na_2_CO_3_, pH 9.5, 6.7 mM/L P-NPP, and 2 mM/L MgCl_2_) was added. The plates were read at 405 nm with a Model 680 microplate reader (Bio-Rad, Hercules, CA, USA). In the assay, baicalein was used as the positive control.

The IC_50_ values of the samples were calculated based on the assay results after curve fitting according to a non-linear regression.

## 4. Conclusions

Six new compounds, including four new lignans dipsalignan A (**1**), B–D (**3**–**5**), and two new bis-iridoid glycoside dimmers, dipsanosides M (**7**) and N (**8**), together with one known compound (**2**), together with two known compounds, (+)-8-hydroxy-7,7'-bis-*epi*-pinoresinol (**2**) and (+)-1, 5-dihydroxy-2(S), 6(*S*)-di(4-hydroxy-3-methoxyphenyl)-3,7-dioxabicyclo[3.3.0] octane (**6**), have been isolated from the roots of *Dipsacus asper* Wall. Compounds **1**, **2**, **7**, and **8** displayed weak HIV-1 integrase inhibitory activities.
